# Full genome sequence of bovine alphaherpesvirus 2 (BoHV-2)

**DOI:** 10.1007/s00705-020-04895-x

**Published:** 2020-12-14

**Authors:** Florian Pfaff, Antonie Neubauer-Juric, Stefan Krebs, Andreas Hauser, Stefanie Singer, Helmut Blum, Bernd Hoffmann

**Affiliations:** 1 Institute of Diagnostic Virology, Friedrich-Loeffler-Institut, 17493 Greifswald - Insel Riems, Deutschland; 2 Bavarian Health and Food Safety Authority, 85764 Oberschleißheim, Germany; 3 Gene Center, Laboratory for Functional Genome Analysis, Ludwig-Maximilians-Universität München, 81377 Munich, Germany

## Abstract

We present the complete genome sequence of bovine alphaherpesvirus 2 (BoHV-2), a member of the family *Herpesviridae*, subfamily *Alphaherpesvirinae*, genus *Simplexvirus*. BoHV-2 is the causative agent of bovine ulcerative mammillitis (bovine herpes mammillitis) and pseudo-lumpy skin disease. The genomic architecture of BoHV-2 is typical of most simplexvirus genomes and congruent with that of human alphaherpesvirus 1 (HHV-1). The genome comprises a total of 131,245 base pairs and has an overall G+C content of 64.9 mol%. A total of 75 open reading frames are predicted. The gene repertoire of BoHV-2 is analogous to that of HHV-1, although the coding region of US12 is missing. A phylogenetic analysis supported BoHV-2 as a member of the genus *Simplexvirus*.

Infection with bovine alphaherpesvirus 2 (BoHV-2), also referred to as bovine mammillitis virus or Allerton virus, causes acute viral diseases in cattle that may develop two separate syndromes: ulcerative mammillitis (bovine herpes mammillitis) or pseudo-lumpy skin disease [1–3]. Pseudo-lumpy skin disease is a generalized form of BoHV-2 infection that causes mild fever and distinct nodular lesions of the skin, comparable to those present during lumpy skin disease [4, 5]. The more common manifestation of BoHV-2 infection is a localized ulcerative disease of the teats and udder of dairy cows or the muzzle of suckling calves [6]. Like other alphaherpesviruses, BoHV-2 establishes latency and may reactivate following primary infection [7]. The genomic architecture of BoHV-2 was initially resolved using restriction endonuclease maps, suggesting a genetic makeup similar to that of human alphaherpesvirus 1 (HHV-1) [8, 9]. Sequence comparison of glycoprotein B (gB) from BoHV-2 with HHV-1 further strengthened their close evolutionary relationship [10], and phylogenetic analysis was finally used for classification of HHV-1 and BoHV-2 as members of the same taxonomic group, which was later named genus *Simplexvirus* [11]. Currently, epidemiological data are unsatisfactory for BoHV-2, phylogenetic analysis is mainly based on partial sequences of gB, and no full-length BoHV-2 genome sequence is available.

In order to facilitate further insights into the genomics and phylogeny of BoHV-2, we selected two BoHV-2 isolates from Germany, stored at the Riems Virus Bank (RVB) of the Friedrich-Loeffler-Institut (Germany), for whole genome sequencing. Strain “Riems 8/85” (collection code RVB-0064) was isolated in 1985 from a cow with mammillitis, while strain “C1Z FZR” (collection code RVB-0062) was isolated in 1986 from a cow with unknown diagnosis.

Both isolates replicated with comparable growth kinetics in bovine esophagus cells (KOP-R) and produced large viral plaques (data not shown). For sequencing, both isolates were propagated on bovine kidney cells (MDBK) until a prominent cytopathic effect was observed. DNA was then isolated from the cell cultures using a MasterPure DNA Extraction Kit (Biozym, Germany). Extracted DNA was then used for Illumina 150-bp paired-end sequencing, resulting in about 6.4 and 7.4 million read pairs for C1Z FZR and Riems 8/85, respectively. Additionally, DNA was used for Oxford Nanopore sequencing, resulting in 80,898 and 3172 reads for C1Z FZR and Riems 8/85, respectively. Illumina raw reads were quality trimmed using TrimGalore (version 0.6.4_dev), and host reads were subtracted using Bowtie2 (version 2.3.5.1) with the *Bos taurus* reference genome ARS-UCD1.2 (GCF_002263795.1). Using this approach, about 92% of read pairs were considered host-derived and not used in further steps. A hybrid assembly of non-host associated-read pairs together with the Nanopore long reads was conducted using Unicycler (version 0.4.8) [12] and SPAdes (version 3.14.0) genome assembler. The resulting scaffolds were combined and arranged with respect to known herpesvirus genomes and the genomic structure for BoHV-2 proposed by Buchman and Roizman in 1978 [9]. For Riems 8/85 a single scaffold contained the non-redundant viral genome sequence, as shown by sequence comparison to HHV-1 and leporid alphaherpesvirus 4 (LeHV-4), and was further used as a guide for the construction of the genome sequence of strain C1Z FZR. Additional mapping and assembly steps were carried out using Newbler (version 3.0) in order to identify and resolve misassemblies of repeats and duplications within the scaffolds of the initial *de novo* assembly, as described previously [13]. Remaining gaps were subsequently closed using PCR and Sanger sequencing. The genomic termini were identified using the software PhageTerm (version 1.0.12) [14] and visual inspection of read depth distributions. Open reading frames (ORFs) in both genomes were annotated using Geneious Prime (version 2019.2.3) in combination with BLASTx and BLASTp (version 2.9.0+). Potential splice sites for genes encoding UL15 and RL2 (ICP0) were predicted using NNSPLICE (version 0.9) [15] and Alternative Splice Site Predictor [16]. The full-length annotated genome sequences of BoHV-2 strains C1Z FZR and Riems 8/85 were uploaded to the DDBJ/EMBL/GenBank databases using accession numbers MT862163 and MT862164, respectively.

The BoHV-2 genome, represented by strain C1Z FZR, is 131,245 nt in length and has an overall G+C content of 64.9 mol%. The unique long (U_L_) and unique short (U_S_) regions comprise 101,047 nt and 12,306 nt with a G+C content of 63.3 mol% and 60.7 mol%, respectively. Both unique regions are flanked by inverted repeats (Fig. 1). The terminal/internal repeat long (T/IR_L_) and terminal/internal repeat short (T/IR_S_) are 3,630 and 5,408 nt in length, respectively, and both have a G+C content of about 77 mol%. The predicted ‘a’ sequence is 184 nt in length and present three times within the genome: two copies are located at both genomic termini, and a single inverted copy is shared between IR_L_ and IR_S_. While BoHV-2 genomic segments U_L_ and U_S_ are of comparable length to their counterparts in HHV-1, the T/IR_S_ and T/IR_L_ repeats are comparably short, which is in accordance with previous results [9].

**Fig. 1:**
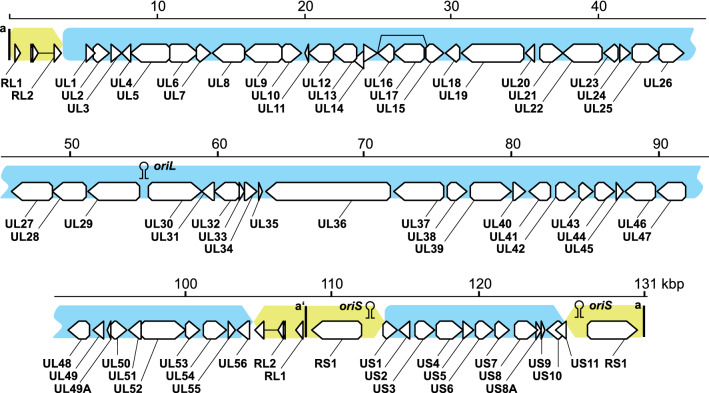
Organization of the BoHV-2 genome, represented by strain C1Z FZR. The unique genomic regions U_L_ and U_S_ (blue arrows) are enclosed by tandem repeats T/IR_L_ and T/IR_S_ (yellow) and the ‘a’ sequence (black bar). Three replication initiation sites (origin of replication—*ori*) and 75 open reading frames (white arrows) have been annotated.

In the ‘a’ sequence, the conserved cleavage and packaging motifs *pac*1 and *pac*2 were identified as a poly(C) rich sequence (poly(C)-GCCGCGAGA-poly(C)) and a poly-A stretch, respectively, within a 91-bp distance of each other and in close proximity to the predicted genomic termini. However, the ‘a’ sequence of BoHV-2 is distinctive, as *pac*1 and *pac*2 appear to be in reverse complement orientation when compared to other simplexviruses (genomic terminus – *pac*1’ – *pac*2’). Furthermore, three origins of replication (*ori*) were detected within the BoHV-2 genome; *oriL* is located between UL29 and UL30, and *oriS* in each copy of T/IR_S_, is located between the RS1 ORF and the respective boundary of the U_S_ region. This arrangement is principally in accordance with that of other simplexviruses; however, *oriL* and *oriS* of BoHV-2 consist of a direct repeat of two stem-loop palindromes that are similar to those observed in macacine herpesvirus 1 [17].

The overall genomic makeup was identical in both BoHV-2 isolates. However, two duplication events were observed in the U_L_ region of the Riems 8/85 genome when compared to C1Z FZR, resulting in two identical copies of UL3 and a duplication located in an untranslated region upstream of UL1. The pairwise nucleotide sequence identity of both BoHV-2 isolates was 95.9% and 98.7% for an alignment of the non-redundant genome including all duplications (125,260 nt) and an alignment excluding poorly aligned positions (121,635 nt), respectively. Both genomes encode 72 non-redundant proteins (from 75 predicted ORFs) that share distinctive sequence identity with proteins from other simplexviruses (Fig. 1). In detail, 60 of the 72 non-redundant proteins from BoHV-2 showed the highest similarity to proteins from LeHV-4 based on BLASTp analysis. BoHV-2 lacks the equivalent of a gene encoding US12 (ICP47), which is present in HHV-1 and is involved in host immune suppression by interfering with antigen presentation [18]. As US12 is mainly present in simplexviruses with primate hosts [13], it may represent a specific adaptation to primates.

A phylogenetic analysis based on the amino acid sequences of 10 herpesvirus core genes from a total of 43 alphaherpesviruses confirmed that BoHV-2 is a member of the genus *Simplexvirus* and that its closest relative is LeHV-4 (Fig. 2). The genomes of BoHV-2 and LeHV-4 show 61% overall nucleotide sequence identity and have a nearly identical genome structure, suggesting a relatively close evolutionary origin in comparison to other alphaherpesviruses. Interestingly, the gene encoding US12 is absent in BoHV-2 and LeHV-4, further supporting a close common ancestry of these viruses.

**Fig. 2:**
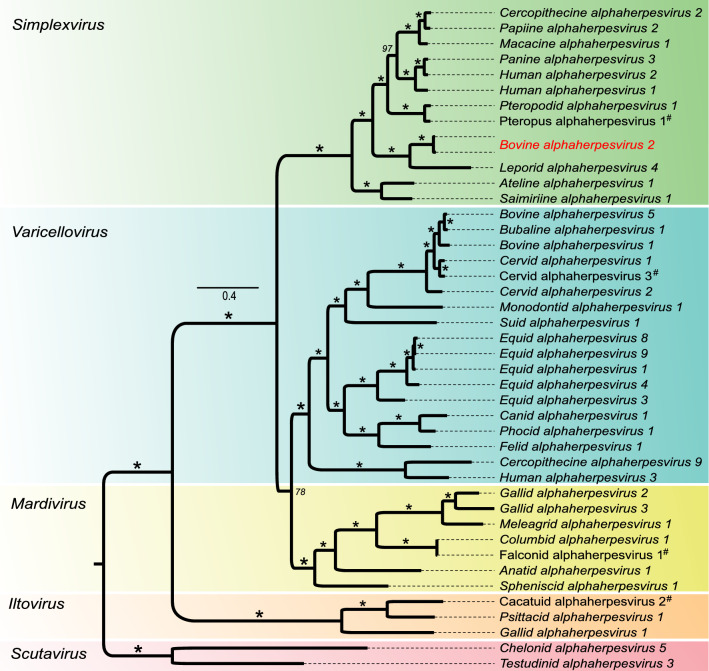
Maximum-likelihood analysis of 43 alphaherpesviruses. The phylogenetic tree was calculated based on an alignment of the concatenated amino acid sequences of the products of the highly conserved herpesvirus core genes UL5, UL9, UL18, UL19, UL27-UL31 and UL33. Scutaviruses were used as an outgroup. Software: IQ-TREE (version 1.6.12; 50.000 ultrafast bootstrap replicates; optimal substitution model for each gene partition). The bar represents amino acid substitutions per site, and an asterisk indicates bootstrap support of 100%, while italic numbers indicate lower bootstrap support. Herpesviruses that are currently not classified at the species level by the ICTV are indicated by a hash symbol

In conclusion, the gene content and genomic arrangement of BoHV-2 are typical of simplexviruses with a class E genome. Phylogenetic analysis suggests that LeHV-4 is currently the closest known relative of BoHV-2. The availability of the full genome sequence of BoHV-2 may ultimately support epidemiological and phylogenetic studies as well as future diagnostic approaches.

## Data Availability

The annotated genome sequences generated during and/or analysed during the current study are available in the DDBJ/EMBL/GenBank databases under the accession numbers MT862163 and MT862164. Additional metadata and raw sequencing data are available under the BioProject ID PRJNA662034.
